# A Kazal-Type Serine Protease Inhibitor from the Defense Gland Secretion of the Subterranean Termite *Coptotermes formosanus* Shiraki

**DOI:** 10.1371/journal.pone.0125376

**Published:** 2015-05-15

**Authors:** Horia Negulescu, Youzhong Guo, Thomas P. Garner, Octavia Y. Goodwin, Gregg Henderson, Roger A. Laine, Megan A. Macnaughtan

**Affiliations:** 1 Department of Chemistry, Louisiana State University and A&M College, Baton Rouge, LA 70803, United States of America; 2 Department of Biological Sciences, Division of Biochemistry and Molecular Biology, Louisiana State University and A&M College, Baton Rouge, LA 70803, United States of America; 3 Department of Entomology, Louisiana State University Agricultural Center, Baton Rouge, LA 70803, United States of America; 4 Currently at the Department of Biochemistry and Molecular Biophysics, Columbia University, New York, NY 10032, United States of America; 5 Currently at the Department of Biochemistry, Albert Einstein College of Medicine of Yeshiva University, Bronx, NY 10461, United States of America; Onderstepoort Veterinary Institute, SOUTH AFRICA

## Abstract

*Coptotermes formosanus* is an imported, subterranean termite species with the largest economic impact in the United States. The frontal glands of the soldier caste termites comprising one third of the body mass, contain a secretion expelled through a foramen in defense. The small molecule composition of the frontal gland secretion is well-characterized, but the proteins remain to be identified. Herein is reported the structure and function of one of several proteins found in the termite defense gland secretion. TFP4 is a 6.9 kDa, non-classical group 1 Kazal-type serine protease inhibitor with activity towards chymotrypsin and elastase, but not trypsin. The 3-dimensional solution structure of TFP4 was solved with nuclear magnetic resonance spectroscopy, and represents the first structure from the taxonomic family, Rhinotermitidae. Based on the structure of TFP4, the protease inhibitor active loop (Cys^8^ to Cys^16^) was identified.

## Introduction

Termite activity has an annual economic impact of over 1 billion dollars in the United States [1,2]. This impact is caused by termite consumption of wood fiber (from live trees and wood structures), crops, plants, and other materials, which contain cellulose. The most destructive and economically important genera are *Coptotermes* and *Reticulitermes* of the subterranean termite family, Rhinotermitidae. Members of the imported Coptotermitidae subfamily are very active in the southern states [1,3].

A termite colony contains a soldier caste, which uses its frontal defense gland secretion and pincer-modified mandibles as weapons to protect the colony. In a *Coptotermes formosanus* colony, soldiers comprise 5–28% of the entire colony and up to 60% of its foraging members [4–6]. The defense gland is well developed in Rhinotermitidae and Termitidae (desert termite) and is a cephalic organ that opens through a frontal pore or fontanelle [7]. The gland reaches deep into the abdominal cavity, and the secretion can comprise as much as one third of the total body weight [8]. During combat, the milky defense secretion of its frontal gland is ejected through a frontal pore onto the attacker, rapidly stiffening in the air [7]. Although much work has been done to determine the chemical composition of the frontal gland secretion from different families of termites, the protein components of the secretions is relatively overlooked.

Henderson, Laine, and others have identified various components of the frontal gland secretion in Coptotermitinae, including n-alkanes, mucopolysaccharide [9], naphthalene [10], free fatty acids, hexacosanoic acid and lignoceric acid [11], and novel ceramides [12]. Recent work has generated interest in caste-specific gene expression. SOL1 is a protein that is found only in the mandibular glands of *Hodotermopsis japonica*, but not in any other stages of differentiation into presoldiers [13]. Ntsp1 is a secretory carrier protein present only in the epithelial cells of the frontal gland reservoir of *Nasutitermes takasagoensis* soldiers [14]. In *Reticulitermes flavipes*, the most common termite in the United States, Scharf *et al*. describe the presence of soldier-specific gene expression of transcription and translation factors with significant sequence homology to the *bicaudal* and *bric-a-brac* genes in Drosophila, which function in embryonic pattern formation [15]. During the research, seven proteins were identified using polyacrylamide gel electrophoresis and amino acid sequencing in the defensive secretion of the frontal gland of *C*. *formosanus* soldiers. Two proteins had lipocalin homologies. A third, novel protein, named TFP4, has homology to Kazal-type serine protease inhibitors and, due to its potential for determining activity and its small size, is amenable to NMR analysis; it is the first of the secretion proteins to be characterized.

**Fig 1:**
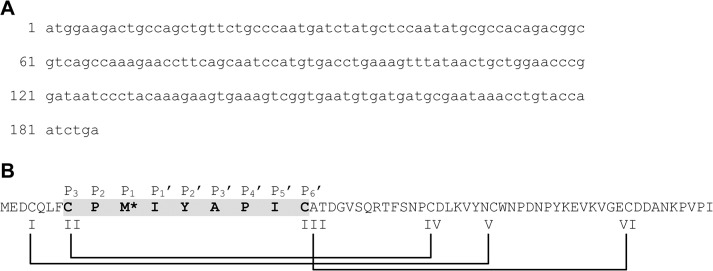
The (A) DNA and (B) primary amino acid sequence of TFP4 cloned from the defense gland secretion of *C. formosanus* termite soldiers. The Kazal-type serine protease inhibitor consensus sequence is highlighted in grey with the active site residue, P_1_, indicated with the asterisk at Met^10^. Laskowski and Kato’s nomenclature for the Kazal-type active loop is shown above the amino acid sequence [21]. Cystines and disulfide bonds are indicated by Roman numerals and lines.

Kazal-type serine protease inhibitors are small proteins, 40–60 amino acids in length, with a structural fold constrained by three disulfide bonds arranged in the order: I-V, II-IV, and III-VI [16,17]. Kazal-type protease inhibitors are classified as “classical” or “non-classical” (group 1 or group 2) based on the relative positions of cystines I and V in the sequence [18,19]. The structures also contain a central α-helix, three-strand anti-parallel β-sheet, and reactive site loop between cystines II and III [16]. The reactive site loop is exposed from the protein's globular structure and adopts a conformation that is complementary to the protease surface [20]. For most classical and non-classical Kazal-type protease inhibitors, the reactive loop, including cystines II and III, is comprised of 9 amino acid residues with little consensus (S1 Fig) [17]. Using nomenclature proposed by Laskowski, *et al*. [21], the third amino acid in the loop sequence, P_1_, is the reactive site residue of the protease inhibitor and determines the enzyme’s specificity (Fig 1). Kazal-type serine protease inhibitors with lysine and arginine at the P_1_ site inhibit trypsin-like enzymes; those with tyrosine, phenylalanine, leucine and methionine inhibit chymotrypsin-like enzymes; and those with alanine, serine, leucine, and methionine at the reactive P_1_ site inhibit elastase-like enzymes [22,23]. The reactive site is typically encompassed by at least one disulfide bridge [24]. Prolines at residues P_2_ and P_4_’ help to ensure proper reactive site geometry, but are not necessary [21]. The Kazal-type protease inhibitors have a similar structural fold, but little sequence consensus, affording the possibility of dual-functionality. Here are presented the activity assays and 3-dimensional structure of TFP4. To our knowledge, TFP4 is the first protein characterized from the termite defense gland secretion

## Materials and Methods

### Crude protein extract collection

Between 150–200 healthy *C*. *formosanus* soldiers were collected from colonies in Brechtel Park, New Orleans, Louisiana. No specific permissions or field permits were required for this location and activity, and the field studies did not involve endangered or protected species. The reason no specific permissions were required is because termites are not a desired insect to have in parks where they are damaging trees. *Coptotermes formosanus* is an invasive species that was brought over during WWII as ships in the Pacific transported infested materials from Japanese islands to port cities in South Carolina, Texas, and Louisiana. They are native to China where they remain the most important pest of wood structures and live trees. Collections of termites have been made from Brechtel Park in New Orleans since 1999. A crate full of wood is buried in the ground near a heavily infested tree and brought to the lab after a month containing up to 30,000 individuals at a time. The termites are contained with food until needed for experiments. The frontal defense gland secretion was isolated on ice by squeezing the head near the frontal pore with tweezers. The secretion was transferred into 5 ml of buffer (10 mM phosphate, 0.15 M sodium chloride, pH 7.0) and centrifuged at 5000 x g for 10 min. The supernatant fraction was filtered (2 μm), dialyzed for 48 hr (3000 Da molecular weight cut-off), and lyophilized.

### Isolation of total mRNA from *C*. *formosanus* soldiers and cDNA library construction

One gram of soldier tissue was prepared for isolation of total mRNA using the Fast Track 2.0 kit (BD Biosciences Clontech) according to the protocol provided by the manufacturer. For the construction of the cDNA library, the BD SMART (Switching Mechanism At 5’ end of RNA Transcript) RACE (Rapid Amplification of cDNA Ends) kit for cDNA synthesis from BD Biosciences Clontech was used by following the manufacturer’s protocol. Oligonucleotides used for the cDNA library construction include: RT reaction primer: 5’-AAGCAGTGGTATCAACGCAGATAC(T)_30_VN-3’ (V = A, G, or C), and cDNA library construction primers: 5’-GAYTGBCARCTNTTYTGBCCNATG-3’ (where Y = T or C, B = T, C, or G; R = A or G), 5’-CTAATACGACTCACTATAGGGCAAGCAGTGGTATCAACGCAGAGT-3’, 5’-CTAATACGACTCACTATAGGGC-3’ (where V = A, G or C). PCR reactions were performed using a thermal cycler, and PCR purification was performed using the QIAquick PCR Purification kit using the reagents and protocol provided.

The purified PCR product was sequenced by the GeneLab sequencing facility at Louisiana State University. A search of the public nucleotide and protein database was performed using the BLAST software [25,26] provided by the National Center for Biotechnology Information. MUSTER (Multi-Sources ThreadER) [27] was used to identify template structures from the Protein Data Bank (PDB) [28] using the TFP4 amino acid sequence. The output from MUSTER includes full-length models of TFP4 built by MODELLER [29] using the template alignments. The threaded structures were visualized with UCSF Chimera software [30].

### Cloning and protein expression

The cDNA of interest was cloned using the pET46Ek/LIC system (Novagen), which provided an *N*-terminal 6X-His tag followed by an enterokinase site (MAHHHHHHVDDDDKP). Oligonucleotides used: 5’-GACGACGACAAGATAGAAGACTGCCAGCTGTT-3’ and 5’-GCTACACCAGCAACATCAACCGGGATTCTCCTC-3’. The recombinant plasmid was transformed into *E*. *coli* BL21(DE3) cells from Novagen for protein expression. The cells were grown in LB media to produce protein for the activity assays and minimal media to produce isotopically labeled protein for NMR analysis. The ^15^N/^13^C-M9 minimal media contained 13 g/L Na_2_HPO_4_, 3 g/L KH_2_PO_4_, 0.5 g/L NaCl, 1 g/L ^15^NH_4_Cl (Cambridge Isotope Laboratories), 2 g/L uniform ^13^C-glucose (Cambridge Isotope Laboratories), 2 mM MgSO_4_, 0.1 mM CaCl_2_, vitamins, and trace metals. The cells were grown at 37°C to an optical density at 600 nm of 0.8, induced with 1 mM isopropyl β-D-1-thiogalactopyranoside, and grown at 37°C for 4 hours. TFP4 was purified from the cleared lysate using nickel or cobalt affinity media and gel filtration chromatography.

### Protease inhibition assays

Elastase (EC 3.4.21.26) and *N*-succinyl-(L-alanine)_3_-p-nitroanilide were purchased from the Sigma-Aldrich Co. Complete Protease Inhibitor cocktail was purchased from Roche. The assays and enzyme activity calculations were performed according to the manufacturer’s protocol, based on the method of Bieth *et al*. [31]. The reactions were monitored at 410 nm at room temperature using a spectrophotometer.

Chymotrypsin (EC 3.4.21.1) and *N*-benzoyl-L-tyrosine ethyl ester (BTEE) were purchased from Worthington Biochemical Corp. and Acros Organics, respectively. The assays to measure enzyme activity and reaction velocity were performed according to the method of Humel *et al*. [32]. The change in light absorbance at 256 nm was monitored at room temperature using a spectrophotometer. The reaction velocity was converted to units of mM/min using the molar extinction coefficient of *N*-benzoyl-L-tyrosine (0.964 mM^-1^min^-1^). The inhibition constant (K_i_) of TFP4 was determined using samples at 50, 75, 100, 200, and 300 μM BTEE with 0, 250, and 500 nM of recombinantly expressed and purified TFP4. Initial velocities (V) at each substrate (S) and inhibitor (I) concentration were analyzed by nonlinear regression analysis using Origin software. The data were globally fitted to competitive, noncompetitive, and uncompetitive inhibition models of which the best model was competitive inhibition (Eq 1). The K_i,_ standard error, and correlation coefficient (R^2^) were determined from the best global fit to Eq 1, where V_max_ is the maximum reaction velocity and K_m_ is the Michaelis-Menten constant.

$$V=\frac{V_{\text{max}}S}{K_{m}(1+\frac{I}{K_{i}})+S}$$ (1)

Trypsin (EC 3.4.21.4) and *N*α-benzoyl-L-arginine ethyl ester were purchased from the Sigma-Aldrich Co. The assays and enzyme activity calculations were performed according to the method described by Bergmeyer *et al*. [33]. Changes in light absorbance were measured at 253 nm at room temperature using a spectrophotometer.

### Protein NMR analysis and structure refinement

All NMR data was collected on a Varian VS-700 MHz spectrometer equipped with a 5-mm HCN-5922 cold probe in the Louisiana State University, Department of Chemistry, NMR facility. ^15^N,^13^C-TFP4 was concentrated to 1 mM in buffer (50 mM potassium phosphate, 50 mM NaCl, 10% deuterated water, pH 6.5) for NMR structure determination. Resonance assignments (^1^H, ^13^C, and ^15^N) were determined using conventional triple-resonance NMR methods [34], the program CcpNmr Analysis [35], and by manual assignment. Chemical shifts have been deposited in the Biological Magnetic Resonance Data Bank (BMRB accession number 18896) [36]. Structure calculations were performed using the program, Crystallography & NMR System (CNS) version 1.2 [37], based on ^1^H-^1^H NOE derived distance constraints calculated from peak volumes using the CcpNmr Analysis program [35], backbone dihedral angle constraints derived from chemical shifts using the programs, CcpNmr Analysis and TALOS+ [38], and disulfide bonds at Cys^4^-Cys^37^, Cys^8^-Cys^30^ and Cys^16^-Cys^52^. The existence of disulfide bonds was confirmed by the ^13^C C^β^ chemical shifts of the oxidized cysteine residues [39]. The configuration of the disulfide bonds, based on other Kazal-type protease inhibitor structures, was verified by NOE constraints between Cys^8^-Cys^30^ and Cys^16^-Cys^52^. Validation of the resulting 10 refined conformers was performed with the Protein Structure Validation Software (PSVS) server version 1.5 [40], including structural statistics and global structure quality factors: Verify3D [41], ProsaII [42], PROCHECK [43], and MolProbity [44]. The final refined ensemble of 10 models and NMR constraint data have been deposited in the PDB (ID 2N17) [28]. Molecular graphics images and protein alignments were produced using the UCSF Chimera software [30]. The average structure, based on the 10 models, was calculated using CNS [10] and analyzed with the Dali server [45] to identify structural homologs in the PDB.

## Results

### Molecular cloning of TFP4 protein from *C*. *formosanus* soldiers defense gland secretion

Sodium dodecylsulfate polyacrylamide gel electrophoresis (SDS-PAGE) of the *C*. *formosanus* soldiers’ defense gland secretion revealed the existence of seven proteins. *N*-terminal sequence determination was performed and one protein, named TFP4, was chosen for further characterization. A cDNA library was constructed using total mRNA from the tissue. Based on the available *N*-terminal sequence, the corresponding cDNA was isolated. The cloned cDNA was sequenced and included a 186 bp reading frame encoding 61 amino acids with a calculated molecular mass of 6888 Da (Fig 1). The nucleotide sequence had no significant alignments using BLAST [25,26], and the protein sequence showed moderate homology to a salivary gland protease inhibitor from *Nauphoeta cinerea*, a cockroach species found in tropical climates. This homology is not surprising since termites and cockroaches descend from a common ancestor. The results showed 40% sequence identity, 54% sequence similarity, and a length of 72 amino acids, similar to TFP4’s 61 amino acids. The cDNA of TFP4 was cloned into a pET46Ek/LIC vector (Novagen) for expression in *E*. *coli*. An *N*-terminal 6X-His tag was included in the recombinant sequence for protein purification (S1 Fig).

### TFP4 is a Kazal-type serine protease inhibitor

Amino acid sequence analysis and threading identified a Kazal-type domain at the *N*-terminus of TFP4 (residues 3–37, Fig 1B). The top five template structures that aligned with the TFP4 sequence using MUSTER (Multi-Sources ThreadER) [27] were all Kazal-type inhibitors: *Anemonia* elastase inhibitor (PDB ID 1Y1B, chain A) [28,46], *Triatoma infestans* factor XIIa inhibitor (infestins 4, PDB ID 2ERW) [47], *Rhodnius prolixus* thrombin inhibitor (rhodniin, PDB ID 1TBQ, chains S and R) [48], *Dipetalogaster maximus* thrombin inhibitor (dipetalin, PDB ID 1KMA) [49], and the third domain of turkey ovomucoid inhibitor in complex with alpha-chymotrypsin (PDB ID 1HJA) [50]. Three of the five structures belong to insect proteins—*T*. *infestans*, *R*. *prolixus*, and *D*. *maximus*—all hematophagous insects with inhibitors to prevent blood coagulation [47–49]. The other two structures from *Anemonia* and turkey are serine protease inhibitors for elastase and alpha-chymotrypsin, respectively [46,50]. The structures predict that TFP4 has a Kazal-type domain with an active loop at amino acids 8–16, Met^10^ at the active site position P_1_ (Fig 1B), an α-helix, a three strand anti-parallel β-sheet, and three disulfide bonds (Cys^4^-Cys^37^, Cys^8^-Cys^30^ and Cys^16^-Cys^52^).

**Fig 2:**
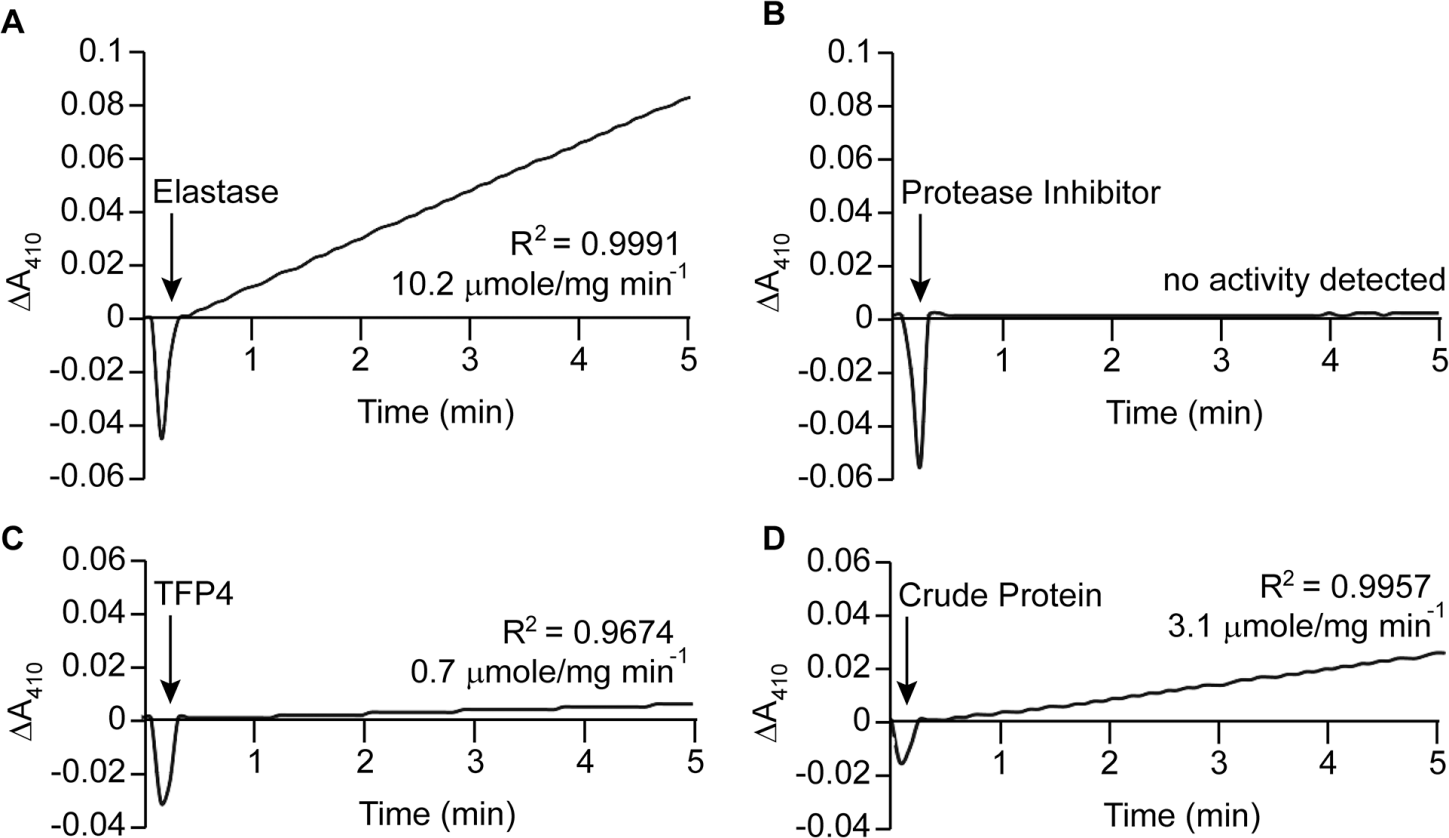
Elastase inhibition assays. (A) Baseline elastase (0.2 μg/mL) activity with *N*-succinyl-(l-alanine)_3_-p-nitroanilide (0.27 mM). Inhibition of elastase with (B) Roche protease inhibitor cocktail (60 μg/mL), (C) TFP4 (2 μg/mL), and (D) crude protein extract (1.2 μg/mL) from the defense gland secretion of *C*. *formosanus*. Additions are indicated by arrows. The hydrolysis rate and correlation coefficient (R^2^) are shown for each reaction.

**Fig 3:**
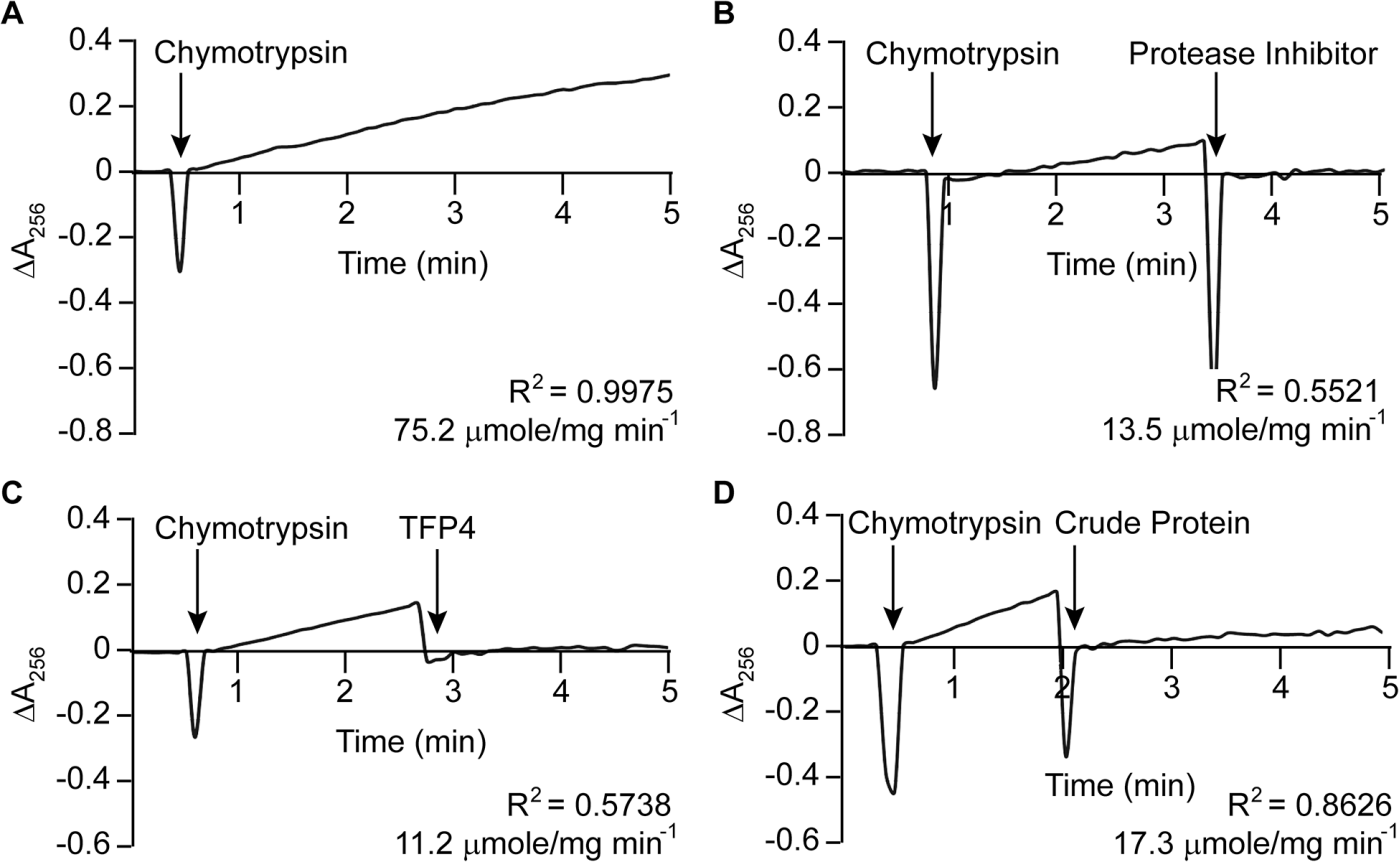
Chymotrypsin inhibition assays. (A) Baseline chymotrypsin (0.1 μg/mL) activity with *N*-benzoyl-L-tyrosine ethyl ester (0.5 mM). Inhibition of chymotrypsin with (B) Roche protease inhibitor cocktail (60 μg/mL), (C) TFP4 (2 μg/mL), and (D) crude protein extract (1.2 μg/mL) from the defense gland secretion of *C*. *formosanus*. Additions are indicated by arrows. The hydrolysis rate and correlation coefficient (R^2^) are shown for each reaction.

**Fig 4:**
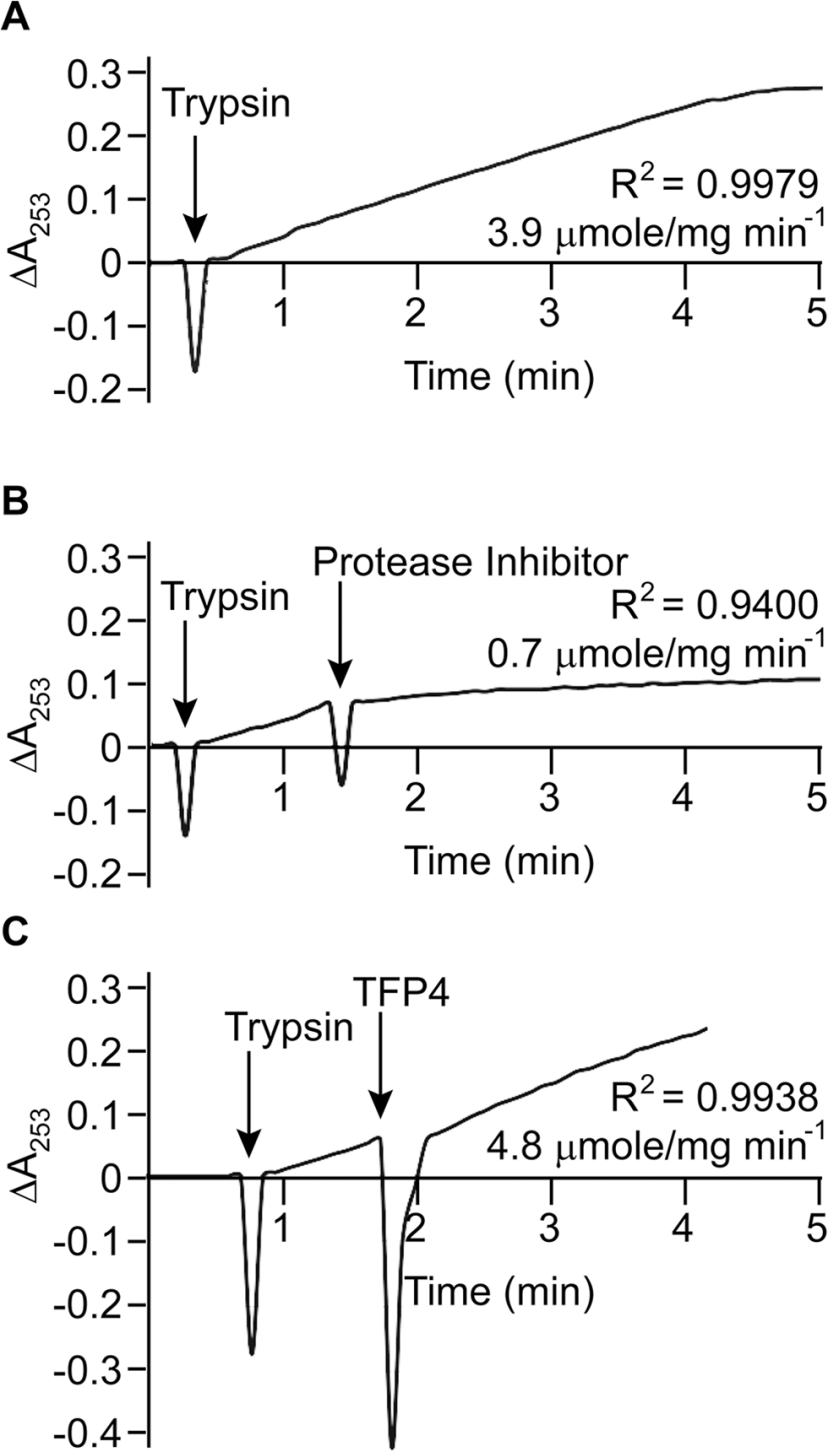
Trypsin inhibition assays. (A) Baseline trypsin (0.3 μg/mL) activity with *N*α-benzoyl-L-arginine ethyl ester (75 μM). Inhibition of trypsin with (B) Roche protease inhibitor cocktail (60 μg/mL) and (C) TFP4 (2 μg/mL). Additions are indicated by arrows. The hydrolysis rate and correlation coefficient (R^2^) are shown for each reaction. The assay with crude extract was not performed since no inhibition was observed with the recombinant protein.

Kazal-type enzymes inhibit serine proteases, a group of proteins which includes trypsin, chymotrypsin, and elastase. TFP4 inhibition was tested by monitoring hydrolysis rates of chromophoric substrates in the presence of each protease. Negative controls of the protease only and positive controls with protease inhibitor cocktail from Roche (60 μg/mL) were performed. Protease inhibition by TFP4 (2 μg/mL) and crude protein extract from the *C*. *formosanus* frontal gland secretion (1.2 μg/mL) were tested with elastase (Fig 2), chymotrypsin (Fig 3), and trypsin (Fig 4). The spectrophotometric traces in Figs 2, 3, and 4 show the hydrolysis rate and correlation coefficient calculated for each reaction. The baseline hydrolysis rates of elastase, chymotrypsin, and trypsin with their respective substrates were 10.2 ± 0.3 (2 replicates), 75.2 ± 1.6 (3 replicates), and 3.9 ± 0.2 (3 replicates) μmoles/mg min^-1^, respectively. As expected, the Roche protease inhibitor cocktail decreased the hydrolysis rate for each protease. Recombinant TFP4 and the crude protein extract showed inhibition of elastase and chymotrypsin, but not trypsin. TFP4 decreased the hydrolysis rate of elastase to 0.7 μmole/mg min^-1^ and chymotrypsin to 11.2 μmole/mg min^-1^. For trypsin, the hydrolysis rate with TFP4 (4.8 μmoles/mg min^-1^) did not change from the baseline rate.

**Fig 5:**
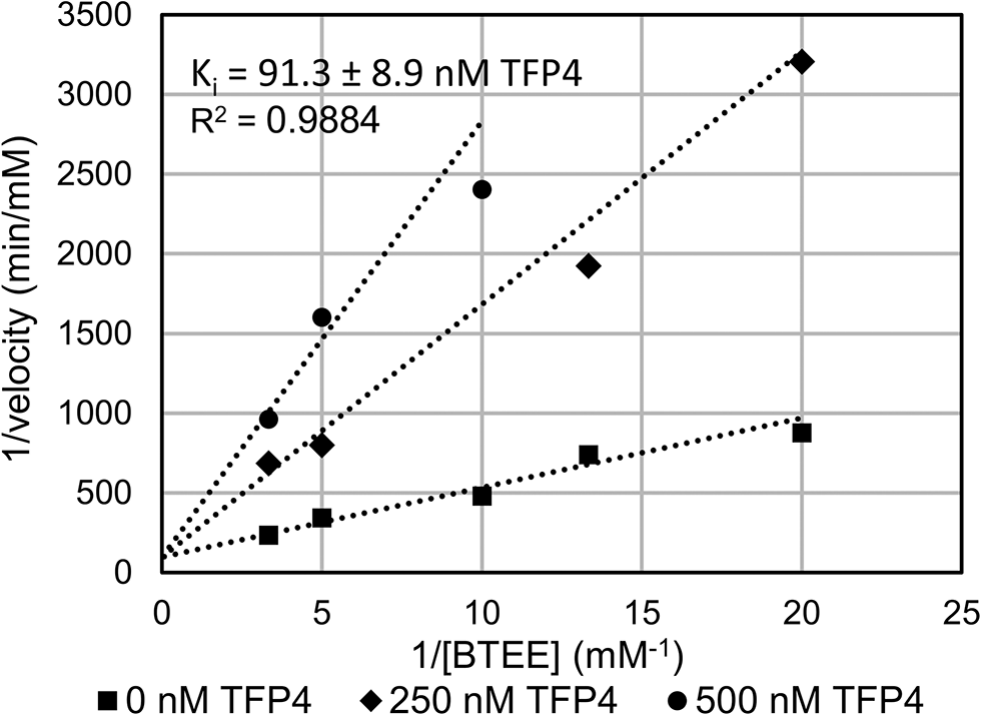
Enzyme kinetic analysis of TFP4 inhibition of BTEE hydrolysis by chymotrypsin. The reaction velocities of chymotrypsin hydrolysis of BTEE at varying concentrations of BTEE and TFP4 are plotted. The points are the observed velocities, and the lines represent the best global fit of the data to Eq 1, a competitive inhibition model. The K_i_ of TFP4 determined from the fit (91.3 ± 8.9 nM) and the correlation coefficient (R^2^ = 0.9884) are displayed on the plot.

The K_i_ of TFP4 for chymotrypsin was determined by measuring the hydrolysis rate of the chromophoric substrate, BTEE, at varying BTEE and TFP4 concentrations. The data shown in Fig 5 were globally fit to a competitive inhibition model to give a K_i_ of 91.3 ± 8.9 nM TFP4. This very strong, competitive inhibition by TFP4 for chymotrypsin is similar to other Kazal-type protease inhibitors [17].

### Solution structure of TFP4 by NMR

**Fig 6:**
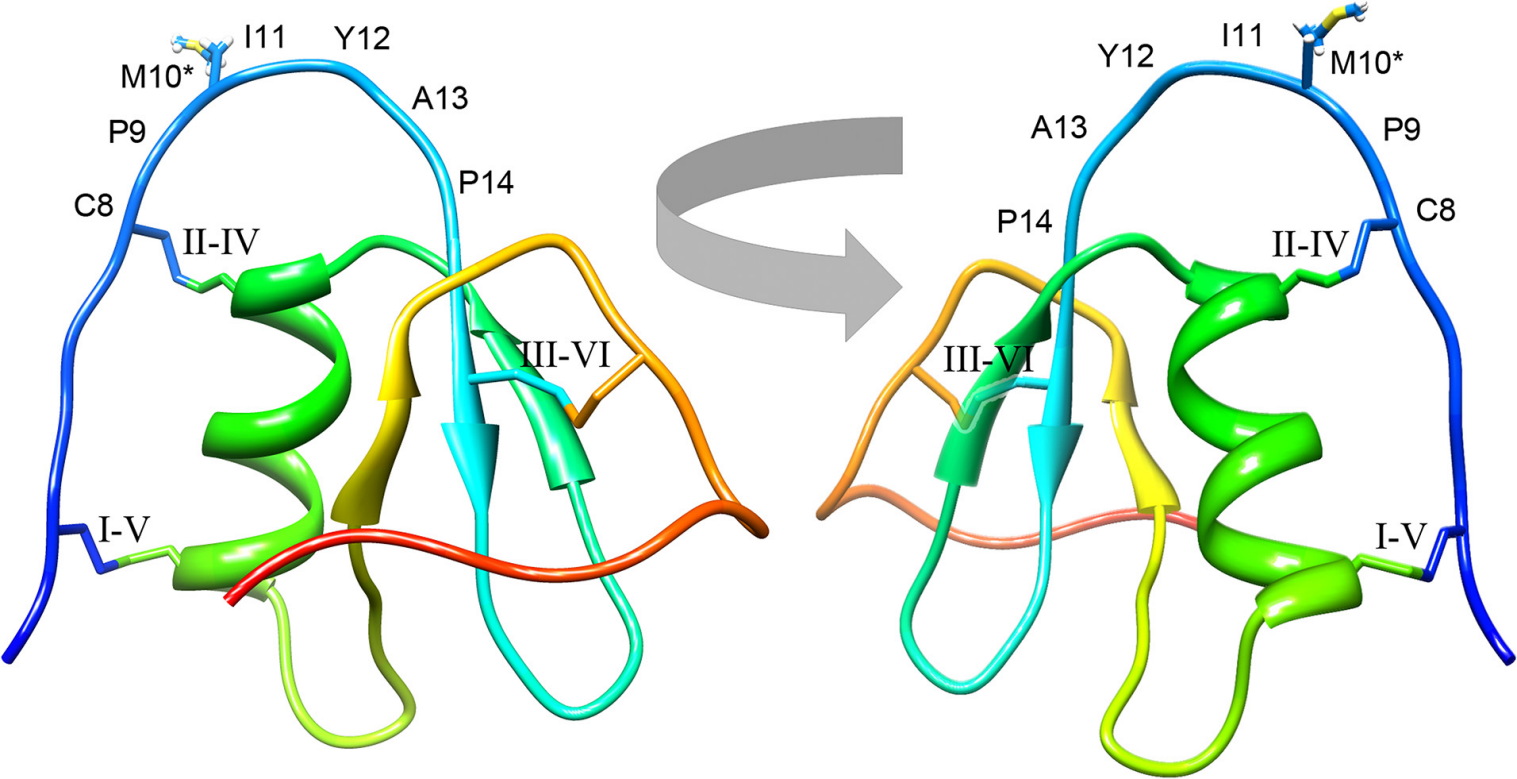
NMR solution structure of TFP4. Two ribbon views of TFP4 are shown to illustrate the Kazal-type consensus loop. The amino acids are numbered based on the TFP4 sequence in Fig 1B. The asterisks indicate the active site amino acid, Met^10^. The secondary structure is colored from the *N*- to *C*-terminus, blue to red. The disulfide bonds are labeled with Roman numerals to indicate the cystine connectivity. Note that partial transparency of the second β-sheet is used in the structure on the right to show the aft III-VI disulfide bond.

The solution NMR structure of TFP4 was determined using triple-resonance NMR and NOESY experiments. An assigned ^1^H,^15^N heteronuclear single quantum coherence spectrum is shown in S3 Fig, and structural statistics are summarized in S1 Table. The structure was determined using 626 constraints including 526 NOE-based distance constraints (100 long range NOE constraints), and 100 dihedral angle constraints [38]. Structure refinement was performed using CNS [37], and the 10 lowest energy structures from 100 structures were selected. The average backbone root mean square deviation (RMSD) of ordered residues in the final ensemble is 0.4 Å (S4 Fig). Backbone dihedral angle analysis indicated that 100% fall in the most favored or allowed regions of the Ramachandran plot [44]. Overall, the global structure quality factors indicate a good, solution structure of TFP4. The average structure in Fig 6 shows an α-helix (Pro^29^-Trp^38^) and a three-strand anti-parallel β-sheet (Ile^15^-Ala^17^, Arg^24^-Phe^26^, Glu^46^-Lys^48^). NOE constraints support the predicted disulfide bond arrangement of Cys^4^-Cys^37^, Cys^8^-Cys^30^, and Cys^16^-Cys^52^. Chemical shifts have been deposited in the Biological Magnetic Resonance Data Bank (BMRB accession number 18896) [36]. The final refined ensemble of 10 models and constraint lists have been deposited in the PDB (ID 2N17) [28].

## Discussion

The amino acid sequence, activity, and structure of TFP4 confirm that it is a non-classical group 1 Kazal-type serine protease inhibitor with activity toward elastase and chymotrypsin, but not trypsin. TFP4 has 61 amino acids and a molecular weight of 6888 Da. The consensus sequence for the active site loop (Cys^8^ to Cys^16^) has a methione (Met^10^) in the reactive P_1_ site, which predicts TFP4’s specificity for chymotrypsin, elastase, and not trypsin [21], corroborating results of the functional assays. The 3D solution structure of TFP4 confirms the non-classical group 1 Kazal-type protease inhibitor structure with three disulfide bonds, a central α-helix, a three-strand anti-parallel β-sheet, and the reactive site loop exposed at the surface (Fig 6) [16]. The position of the disulfide bonds, Cys^4^-Cys^37^, Cys^8^-Cys^30^, and Cys^16^-Cys^52^, classifies TFP4 as a non-classical group 1 Kazal-type with cystines I and V near the *C*-terminus of the α-helix (Fig 6) [46]. Other structural conditions for Kazal-type protease inhibitor activity are met with two disulfide bridges at either end of the reactive loop (Fig 6) [24] and prolines at residues P_2_ and P_4_’ to ensure proper reactive site geometry (Fig 1B) [21].

**Fig 7:**
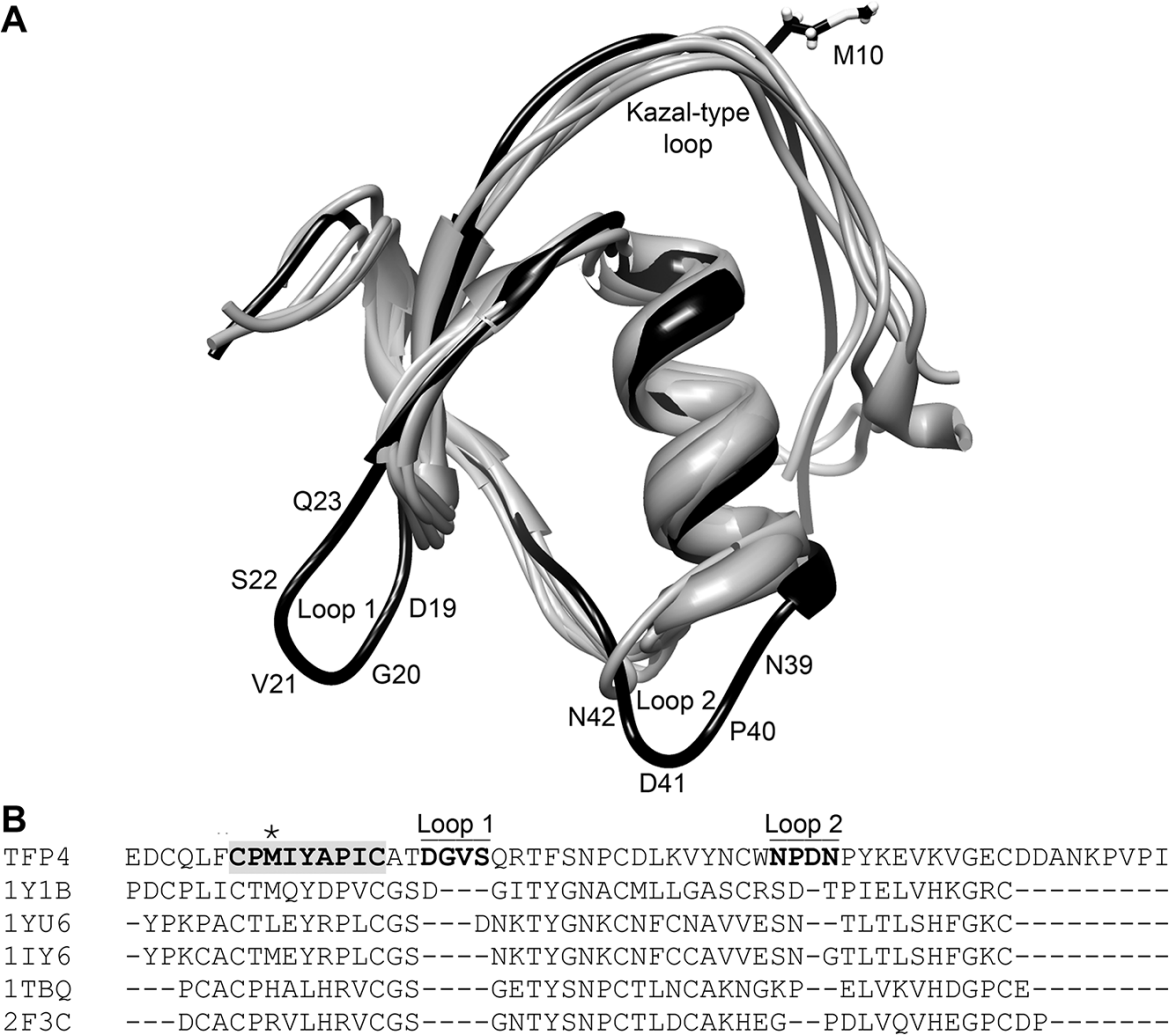
TFP4 structure aligned with structural homologs. (A) The top five, non-redundant, structural homologs as determined by the Dali server [45] aligned with the average TFP4 structure. TFP4 is shown in black and the structural homologs in grey (PDB ID: 1Y1B chain A, 1YU6 chain B, 1IY6 chain A, 1TBQ chain S, 2F3C chain I) [46–48,51,52]. The extra loops in TFP4 that are not found in the homologous structures are labeled, Loop 1 and Loop 2, with their corresponding amino acid sequences. (B) Sequence alignments based on the structural alignment of TFP4 and the homologous proteins were generated using the Dali server [45]. The Kazal-type consensus sequence is highlighted in bold with the active site residue indicated by the asterisk. Loops 1 and 2 are labeled with the corresponding residues are in bold.

The top five, non-redundant, structural homologs of TFP4, as determined by the Dali server [45], are all Kazal-type inhibitors: anemonia elastase inhibitor [46], turkey ovomucoid third domain [51], silver pheasant ovomucoid third domain [52], *Rhodnius prolixus* rhodniin [48], and *Triatoma infestans* infestins [47] (PDB ID: 1Y1B chain A, 1YU6 chain D, 1IY6 chain A, 1TBQ chain S, 2F3C chain I, respectively). A structural and sequence alignment of TFP4 with the homologous structures show loops that are not present in the other Kazal-type protease inhibitors (Fig 7). The sequence between cystines V and VI is not well conserved among Kazal-type protease inhibitors and can vary in lengths from 9–21 amino acids [17]. Thus, the extra loop with Asp^41^ (Loop 2 in Fig 7) is not surprising given the loop can be various lengths. The sequence between cystines III and IV is conserved as determined from 85 invertebrate Kazal-type protease inhibitor sequences [17] using WebLogo v3 [53,54] (S5 Fig). The sequence alignment of TFP4 with the consensus sequence indicates that Ser^22^-Gln^23^-Arg^24^ are extra residues that result in an extended β-hairpin (Loop 1 in Fig 7).

TFP4 is the first protein cloned and characterized from the frontal defense gland secretion of termite soldiers. TFP4’s function is likely to protect the content of the termite defense gland in *C*. *formosanus* from microbial proteolytic enzymes. This gland has contents that comprise one third of the soldier’s mass with a foramen in the head that could conceivably be accessed by bacteria and fungi. The protease inhibitor activity may be antimicrobial as bacteria and fungi produce proteases during the infection process [55,56]. The protease activity may also assist in the protection of the protein content of the defense fluid, whose additional functions remain to be determined. The 3D solution structure of TFP4 is the first structure deposited in the PDB (ID 2N17) from the family Rhinotermitidae (subterranean termites) and only the fourth protein from the order Isoptera (termites) [57–60].

## Supporting Information

S1 Fig The (A) sequence logo for the Kazal-type protease inhibitor active loop and (B) its alignment with the TFP4 sequence. (A) The logo was created using 83 invertebrate Kazal-type protease inhibitors [17] for the amino acid sequence between cystines II and III using WebLogo v.3 [53]. The overall height at each position indicates the relative sequence conservation, and the heights of the symbols indicate the relative frequency of each amino acid. (B) The asterisks indicate agreement between the consensus sequence and TFP4. Laskowski and Kato’s nomenclature for the Kazal-type protease inhibitor sequence is shown [21].
(TIF)

S2 Fig The recombinant TFP4 sequence with a 6X-His tag. The 6X-His tag is underlined with numbering from (-15 to -1). The TFP4 sequence starts at Glu^2^ and is numbered to be consistent with Fig 1B.
(TIF)

S3 Fig An assigned ^1^H-^15^N heteronuclear single quantum coherence spectrum of ^15^N,^13^C-TFP4. Assignments of the amide backbone peaks are indicated by the residue number and single character amino acid code next to each peak. Dashed lines indicate side-chain peaks. Residues are numbered as in the S2 Fig.
(TIF)

S4 Fig Ensemble of 10 NMR structures of TFP4. The 10 lowest energy structures from 100 models were aligned using the PSVS server [40]. Residues Glu^2^-Ile^61^ without the 6X-His tag are shown in ribbon view with the disulfide bonds in black. The RMSD of the ordered residues in the 10 models (Cys^4^-Val^59^, defined by conformationally restricting NMR constraints) is 0.4 Å for the backbone heavy atoms and 0.9 Å for all heavy atoms.
(TIF)

S5 Fig The sequence conservation of (A) the 12 amino acids between cystines III and IV of invertebrate Kazal-type domains compared to (B) the corresponding TFP4 sequence. WebLogo v.3 [53] was used to create the consensus sequences using 85 invertebrate sequences. The overall height at each position indicates the relative sequence conservation, and the heights of the symbols indicate the relative frequency of each amino acid. (B) The asterisks indicate agreement between the TFP4 sequence and the consensus sequences. The periods indicate gaps added to align the two sequences. The dash indicates very low sequence conservation.
(TIF)

S1 Table NMR and structural statistics. Structural statistics were computed using the Protein Structure Validation Software (PSVS) Version 1.5 [40] for the ensemble of 10 NMR structures (S4 Fig) deposited in the PDB (ID 2N17) [28]. All analyses were performed using the ordered residues, Cys^4^-Val^59^.
(DOCX)
