# Supplementary figures and images for: A Kazal-Type Serine Protease Inhibitor from the Defense Gland Secretion of the Subterranean Termite Coptotermes formosanus Shiraki
Source: PLoS One. 2015 May 15;10(5):e0125376. doi: 10.1371/journal.pone.0125376 (PMC4433142; doi:10.1371/journal.pone.0125376)

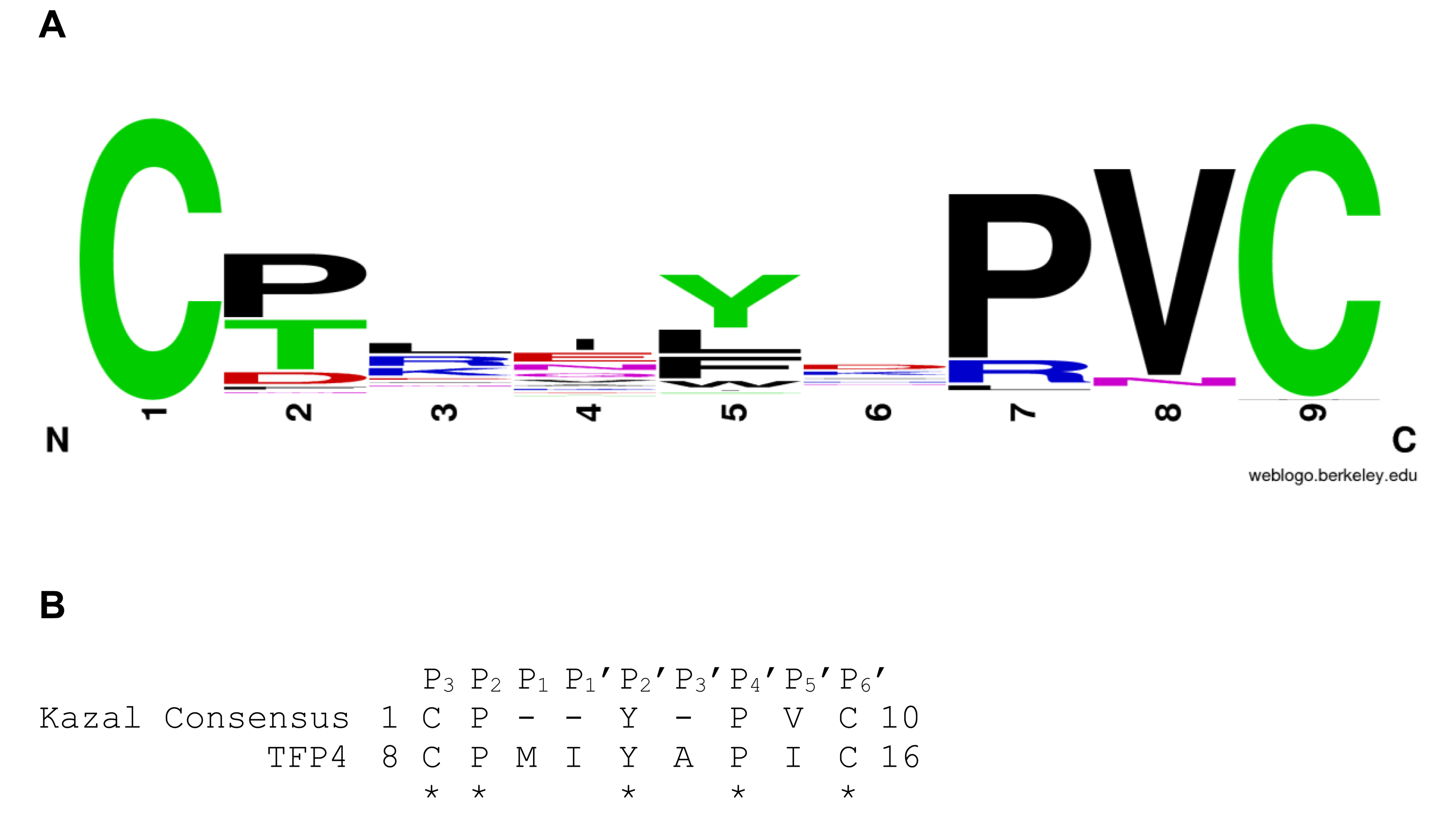

Supplement: S1 Fig — (A) The logo was created using 83 invertebrate Kazal-type protease inhibitors [17] for the amino acid sequence between cystines II and III using WebLogo v.3 [53]. The overall height at each position indicates the relative sequence conservation, and the heights of the symbols indicate the relative frequency of each amino acid. (B) The asterisks indicate agreement between the consensus sequence and TFP4. Laskowski and Kato’s nomenclature for the Kazal-type protease inhibitor sequence is shown [21]. (TIF) [file pone.0125376.s001.tif]

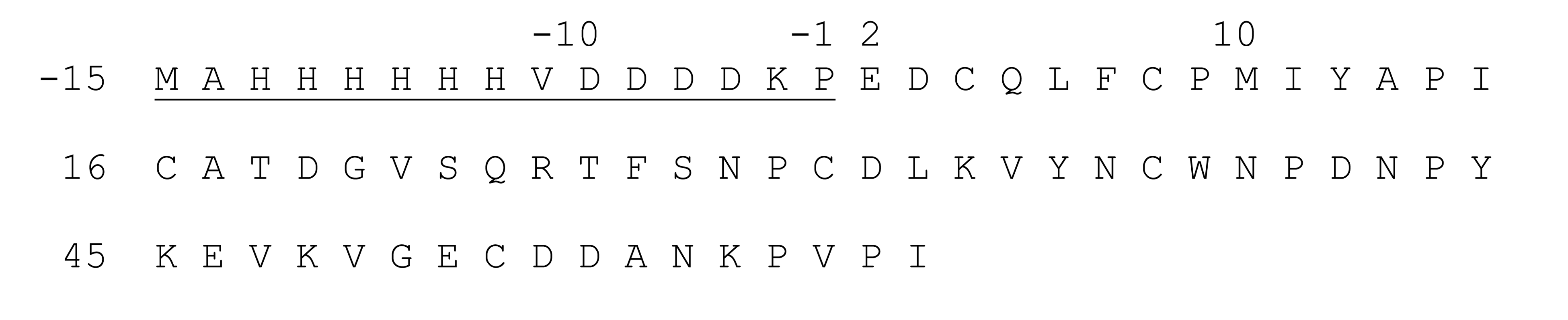

Supplement: S2 Fig — The 6X-His tag is underlined with numbering from (-15 to -1). The TFP4 sequence starts at Glu2 and is numbered to be consistent with Fig 1B. (TIF) [file pone.0125376.s002.tif]

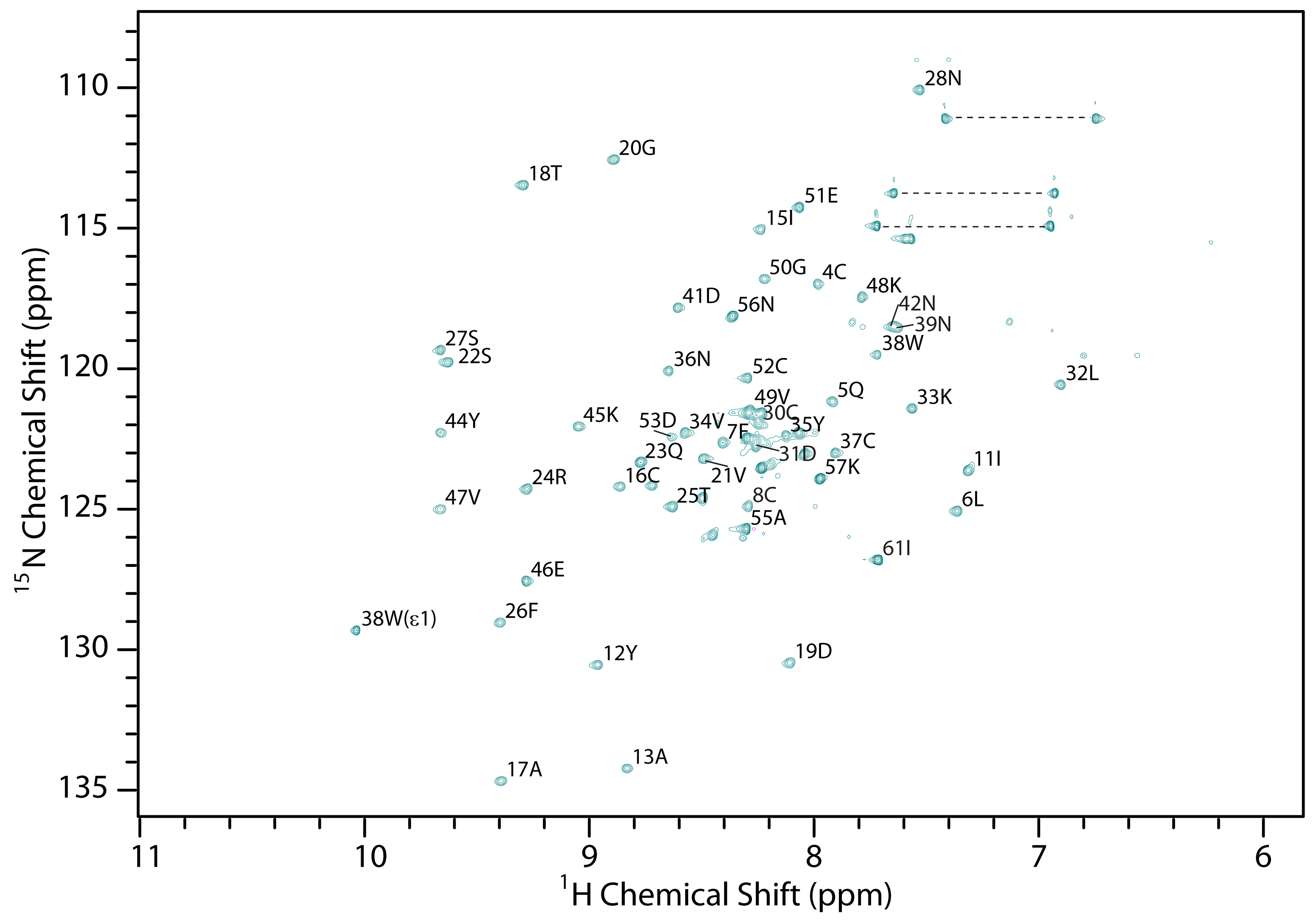

Supplement: S3 Fig — Assignments of the amide backbone peaks are indicated by the residue number and single character amino acid code next to each peak. Dashed lines indicate side-chain peaks. Residues are numbered as in the S2 Fig. (TIF) [file pone.0125376.s003.tif]

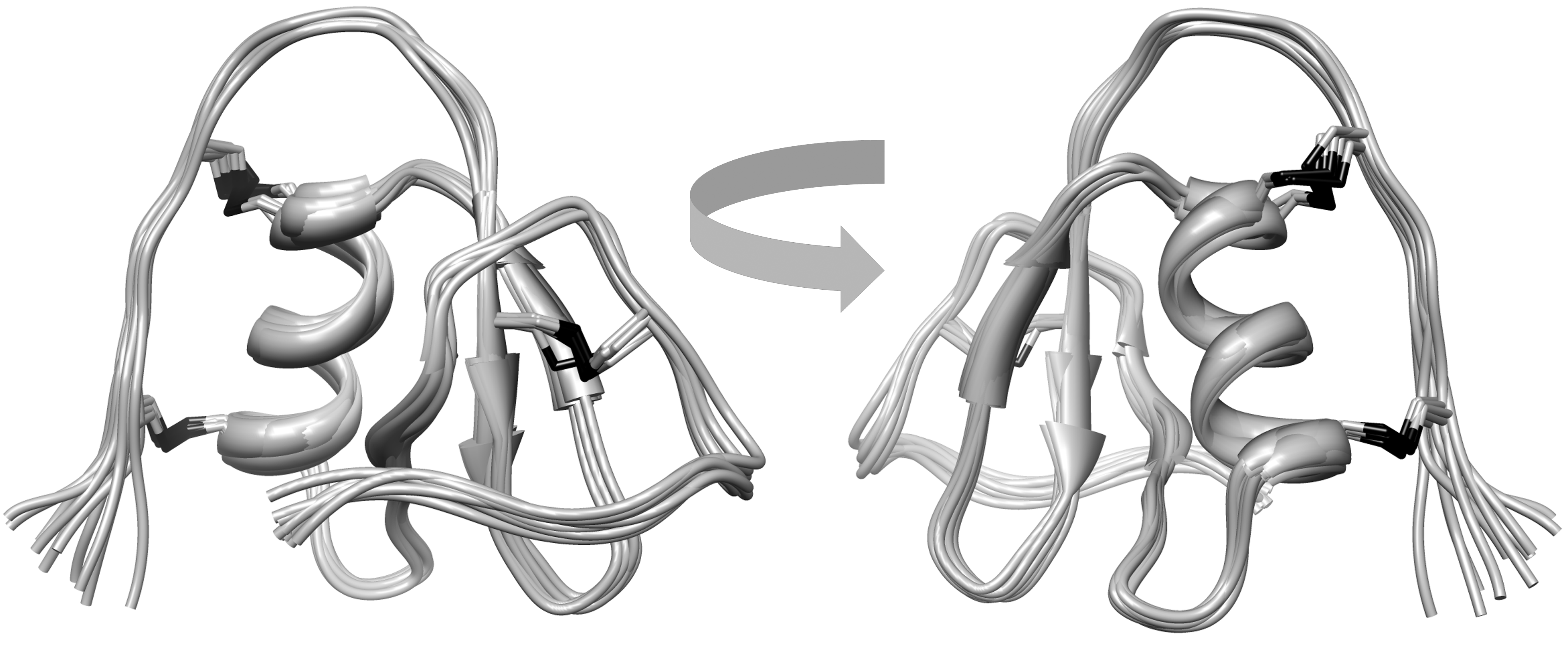

Supplement: S4 Fig — The 10 lowest energy structures from 100 models were aligned using the PSVS server [40]. Residues Glu2-Ile61 without the 6X-His tag are shown in ribbon view with the disulfide bonds in black. The RMSD of the ordered residues in the 10 models (Cys4-Val59, defined by conformationally restricting NMR constraints) is 0.4 Å for the backbone heavy atoms and 0.9 Å for all heavy atoms. (TIF) [file pone.0125376.s004.tif]

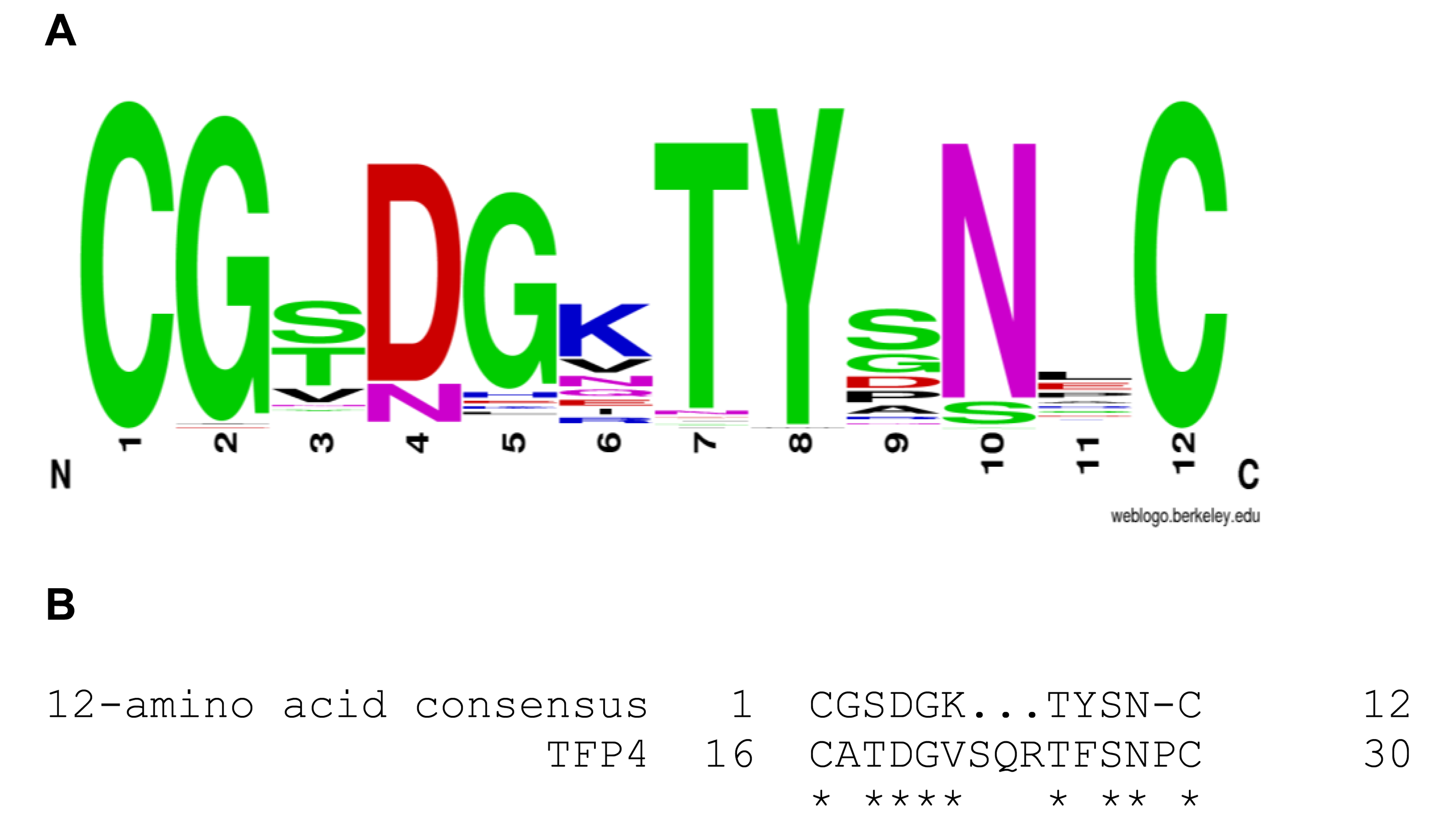

Supplement: S5 Fig — WebLogo v.3 [53] was used to create the consensus sequences using 85 invertebrate sequences. The overall height at each position indicates the relative sequence conservation, and the heights of the symbols indicate the relative frequency of each amino acid. (B) The asterisks indicate agreement between the TFP4 sequence and the consensus sequences. The periods indicate gaps added to align the two sequences. The dash indicates very low sequence conservation. (TIF) [file pone.0125376.s005.tif]
