# Supplementary material for: A Kazal-Type Serine Protease Inhibitor from the Defense Gland Secretion of the Subterranean Termite Coptotermes formosanus Shiraki
Source: PLoS One. 2015 May 15;10(5):e0125376. doi: 10.1371/journal.pone.0125376 (PMC4433142; doi:10.1371/journal.pone.0125376)
Supplement: S1 Table — Structural statistics were computed using the Protein Structure Validation Software (PSVS) Version 1.5 [40] for the ensemble of 10 NMR structures (S4 Fig) deposited in the PDB (ID 2N17) [28]. All analyses were performed using the ordered residues, Cys4-Val59. (DOCX) [file pone.0125376.s006.docx]

**S1 Table.**

| **Total number of constraints** | 626 |
| --- | --- |
| **NOE-based, conformationally-restricting distance constraints** | |
| Total | 526 |
| Intra-residue [  i = j] | 191 |
| Sequential [\|i - j\| = 1] | 185 |
| Medium range [1 < \|i - j\| < 5] | 50 |
| Long range [\|i - j\| ≥ 5] | 100 |
| **Dihedral angle constraints** | 100 |
| **Total number of restricting (long-range) constraints per restrained residue*^a^*** | 11.4 (1.8) |
| **Total structures computed** | 100 |
| **Number of structures used** | 10 |
| **Residual constraint violations*^b^*** | |
| Distance violations per structure |  |
| 0.2 – 0.5 Å | 18.7 |
| > 0.5 Å | 0 |
| RMS of distance violation / constraint (Å) | 0.06 |
| Dihedral angle violations per structure |  |
| 1 – 10° | 18.1 |
| > 10° | 0 |
| RMS of dihedral angle violation / constraint (°) | 1.43 |
| **RMSD (Å) for ordered residues** | |
| Backbone heavy atoms | 0.4 |
| All heavy atoms | 0.9 |
| **Structure quality factors (-raw score/Z-score)** | |
| Procheck G-factor***^c^*** (phi-psi only) | -0.59/-2.01 |
| Procheck G-factor***^c^*** (all dihedral angles) | -0.92/-5.44 |
| Verify3D | 0.12/-5.46 |
| ProsaII (-ve) | 0.05/-2.48 |
| Molprobity clashscore | 21.35/-2.14 |
| **Ramachandran plot summary from Procheck (%)** | |
| Most favored regions | 84.1 |
| Additionally allowed regions | 15.9 |
| Generously allowed regions | 0.0 |
| Disallowed regions | 0.0 |
| **Deviations from Ideal Geometry (from PDB validation software)*^c^*** |  |
| Number of close contacts***^d^*** | 0 |
| RMS deviation for bond angles (°) | 1.2 |
| RMS deviation for bond lengths (Å) | 0.013 |

***^a^*** There are 55 residues with conformationally restricting constraints

***^b^*** Calculated for all constraints using sum over r^-6^

***^c^*** Residues with sum of phi and psi order parameters > 1.8

***^d^*** Within 1.6 Å for H atoms and 2.2 Å for heavy atoms
